# Health worker perceptions of stigma towards Zambian adolescent girls and young women: a qualitative study

**DOI:** 10.1186/s12913-022-08636-5

**Published:** 2022-10-17

**Authors:** Caroline Meek, Drosin M. Mulenga, Patrick Edwards, Sophie Inambwae, Nachela Chelwa, Michael T. Mbizvo, Sarah T. Roberts, Sujha Subramanian, Laura Nyblade

**Affiliations:** 1 Center for Health Analytics, Media, and Policy, RTI International, Washington, DC USA; 2 Department of Health Behavior, Gillings School of Global Public Health, University of North Carolina at Chapel Hill, Chapel Hill, NC USA; 3 Population Council, Lusaka, Zambia; 4 Health Care Financing and Payment Program, RTI International, Research Triangle Park, NC USA; 5 Women’s Global Health Imperative, RTI International, Berkeley, CA USA; 6 Public Health Research Division, RTI International, Waltham, MA USA; 7 Global Health Division, RTI International, Washington, DC USA

**Keywords:** Stigma, HIV, Adolescent girls and young women, Health facility, Sexual and reproductive health

## Abstract

**Background:**

The high prevalence of HIV among adolescent girls and young women aged 15–24 in Eastern and Southern Africa indicates a substantial need for accessible HIV prevention and treatment services in this population. Amidst this need, Zambia has yet to meet global testing and treatment targets among adolescent girls and young women living with HIV. Increasing access to timely, high-quality HIV services in this population requires addressing the intensified anticipated and experienced stigma that adolescent girls and young women often face when seeking HIV care, particularly stigma in the health facility setting. To better understand the multi-level drivers and manifestations of health facility stigma, we explored health workers’ perceptions of clinic- and community-level stigma against adolescent girls and young women seeking sexual and reproductive health, including HIV, services in Lusaka, Zambia.

**Methods:**

We conducted 18 in-depth interviews in August 2020 with clinical and non-clinical health workers across six health facilities in urban and peri-urban Lusaka. Data were coded in Dedoose and thematically analyzed.

**Results:**

Health workers reported observing manifestations of stigma driven by attitudes, awareness, and institutional environment. Clinic-level stigma often mirrored community-level stigma. Health workers clearly described the negative impacts of stigma for adolescent girls and young women and seemed to generally express a desire to avoid stigmatization. Despite this lack of intent to stigmatize, results suggest that community influence perpetuates a lingering presence of stigma, although often unrecognized and unintended, in health workers and clinics.

**Conclusions:**

These findings demonstrate the overlap in health workers’ clinic and community roles and suggest the need for multi-level stigma-reduction approaches that address the influence of community norms on health facility stigma. Stigma-reduction interventions should aim to move beyond fostering basic knowledge about stigma to encouraging critical thinking about internal beliefs and community influence and how these may manifest, often unconsciously, in service delivery to adolescent girls and young women.

**Supplementary Information:**

The online version contains supplementary material available at 10.1186/s12913-022-08636-5.

## Background

In Eastern and Southern Africa, HIV prevalence among adolescent girls and young women (AGYW) aged 15–24 is more than double that of males in the same age group [1]. In addition to an increased biological susceptibility to HIV [2], AGYW face gender-based social and economic power imbalances that make them particularly vulnerable to HIV, in addition to increasing their susceptibility to sexually transmitted infections (STIs) and early pregnancy [3]. Access to high-quality, timely sexual and reproductive health (SRH) services, including HIV prevention and treatment services, is essential to the health and well-being of AGYW and the achievement of global HIV targets. The Joint United Nations Programme on HIV/AIDS (UNAIDS) 90–90-90 targets aimed for 90% of people living with HIV to know their status, 90% of those diagnosed to have initiated antiretroviral therapy (ART), and 90% of people on ART to be virally suppressed by 2020 [4]. Although Zambia was one of the 7 countries in Eastern and Southern Africa that met the 90–90-90 targets [5], the country has not yet reached these targets among the AGYW population living with HIV. Only 40% of Zambian AGYW living with HIV know their status, 78% of these AGYW are on ART, and 78% of the AGYW on ART are virally suppressed [6].

These numbers indicate that more work remains in making SRH services accessible for AGYW in Zambia. Successful promotion of AGYW engagement in HIV testing, care, and treatment involves addressing the particular barriers that AGYW face in accessing SRH services in general, as well as HIV services specifically. These barriers are compounded by morality narratives associated with the unique combination of being young, female, and perceived as sexually active [7]. As a result of this combination, AGYW seeking SRH services face intensified anticipated and experienced stigma stemming from gender-based narratives related to personal blame, shame, immorality, promiscuity, and sex work [7, 8]. The higher risk and increased burden of HIV on AGYW, the shortfall in meeting the 90–90-90 HIV targets for AGYW in Zambia, and the overlapping stigmatizing narratives based on the combination of adolescence, young adulthood, and gender all point to the need for more research into the specific barriers that AGYW face when accessing SRH services.

UNAIDS has acknowledged stigma as a persistent barrier that restricts access to HIV prevention, care, and treatment and formed the Global Partnership for Action to Eliminate All Forms of HIV-Related Stigma and Discrimination in 2017 [9], noting an urgency to focus on AGYW as a group left behind in the HIV response to date. The report discussed the harm of stigma in health care settings [9], reflecting previous evidence that stigma from health workers has a particularly negative impact on testing uptake, care engagement, and treatment adherence [10–13]. In response to the increased recognition of stigma from health workers as an access barrier to HIV prevention and treatment services, as well as other SRH services, several studies have focused on facility-level stigma-reduction interventions in recent years [14–19]. Research has also highlighted the need for multi-level stigma-reduction interventions due to the inherently cross-sectoral nature of stigma, as well as called for further exploration of how changes in stigma at one socio-ecological level affects stigma at other socio-ecological levels [20–22]. Within the current literature, few studies have explored the cross-sectoral drivers of health worker stigma towards AGYW or, more broadly, have focused on interventions to reduce stigma directed towards AGYW among health workers.

In response to this gap, stigma research and interventions are embedded as an integral part of a study developing and testing a multi-level model for HIV prevention and treatment targeting AGYW in Zambia [23]. This article describes the results from formative work conducted to inform integration of health worker AGYW stigma-reduction into the multi-level study, specifically the adaptation of an evidence-based, participatory stigma-reduction training [17] focused on AGYW stigma in health facilities in Lusaka, Zambia. We present the results of the formative work, which aid in understanding the nuances of AGYW stigma across community and clinic levels from the perspective of the health workers themselves. Stigma is a social process deeply rooted in communities, and despite their role as medical professionals, health workers are not separate from the social fabric that makes up their larger community. Understanding the multi-level drivers and manifestations of health worker AGYW stigma is necessary to designing and implementing AGYW stigma-reduction interventions in health facilities, thereby increasing AGYW access to HIV and other SRH services.

## Methods

### Setting

Data were collected from health workers across six public health facilities participating in the parent study [23]. All facilities are in the densely populated, primarily low-income urban and peri-urban areas of Lusaka, Zambia, with an estimated population of 2 million people and 190 public or private health facilities.

### Study sample

We conducted 18 in-depth interviews (IDIs) (15 females/three males), ranging from around 30–90 min, with 14 clinical and four non-clinical staff across the six facilities. Eligibility criteria included being aged 18 or older and working in the study health facilities for at least 6 months in a position, whether clinical or non-clinical, that interacts with AGYW clients. The study team and a Community Advisory Board member identified potential participants by liaising with the leadership from each facility to nominate three clinic staff from different departments who they assessed would be knowledgeable and actively engaged informants. Selected participants included clinic in-charges (heads of departments), community health workers, and adolescent focal point persons, as well as employees from three departments where AGYW are commonly seen—Maternal and Child Health, labor ward, and registration. Duration of respondents’ service in the health sector ranged from 2.5 to 20 years.

### Data collection/management

IDIs were conducted in August 2020 by three experienced Zambian female qualitative interviewers in a private setting using a semi-structured guide developed for the study (Supplementary File 1). IDI topics focused on eliciting health workers’ perceptions of the drivers, manifestations, and impacts of stigma towards AGYW in both the clinic and the community. For example, interviewers asked questions such as ‘Do you feel stigma is an issue for adolescent girls and unmarried young women who are having sex?’ and further probed about what this stigma looked like, who it was coming from, and how this stigma manifested when seeking care at the clinic. Interviewers participated in a 3-day training that included content related to stigma, practice with the interview guides, a refresher on qualitative methods, and research ethics. Within 1 day of each IDI, the interviewer summarized the main findings in a debrief report, which is a short, structured form designed to highlight key concepts [24]. Each debrief report was reviewed by the senior co-investigator leading this sub-study to provide rapid feedback on interviewing techniques and probing approaches. Interviewers engaged in debriefing sessions regularly throughout the data collection period to share and incorporate this feedback. All interviews were conducted in English, audio recorded and transcribed verbatim, and then checked for quality by the field research team lead.

### Analysis

After close reading of transcripts and debrief reports, the research team developed a codebook capturing deductive (i.e., from topics probed in the semi-structured guides) and inductive (i.e., emerging from participant narratives) themes. Two trained analysts applied codes to text segments from IDI transcripts using the Dedoose (2019) web application. At the beginning of coding, each team member independently coded one transcript using the preliminary codebook, and the coding results were compared to reach consensus on how codes should be applied, the adequacy of code definitions, and the completeness of the codebook. The codebook was revised, and this process was repeated with one additional transcript. Coding discrepancies were reviewed and resolved during weekly quality control meetings, where the team also discussed any questions and emerging themes. After each meeting, the codebook was revised to reflect any changes, and coding of previous transcripts was updated as needed to reflect these changes. An inter-rater reliability test of several key concepts resulted in a Cohen’s Kappa score of 0.75, which is considered to indicate substantial agreement between coders [25]. After coding all transcripts, analysts reviewed coded text segments, identifying salient themes and patterns to aid data synthesis and interpretation.

### Ethical clearance

Ethical clearance was provided by three review boards: the ERES Converge Research Ethics Committee in Zambia as well as the Institutional Review Boards at the Population Council and the University of North Carolina-Chapel Hill. Interviewers provided study details and obtained written consent from all participants before initiating the interviews.

## Results

Health workers’ descriptions of stigma in the clinic largely reflected the stigma that they reported observing in the community, an overarching theme that we further explored using the HIV stigma-reduction implementation framework presented by Nyblade et al. [26]. This framework provided a guiding framework for our analysis and focuses on immediately actionable drivers of stigma, manifestations of stigma, and impacts to the HIV care continuum.

### Drivers and manifestations of stigma in clinics: a reflection of community stigma

Three of the framework’s immediately actionable drivers, namely attitudes, awareness, and institutional environment, were observed in the interviews. Health workers also discussed several experienced and perceived manifestations of stigma that arise from each of these drivers; the clinic manifestations largely mirrored the community manifestations.

#### Attitudes as a driver

The first driver, attitudes, refers to the judgment, shaming, and stereotyping involved in stigmatization, often rooted in deeply entrenched religious or cultural beliefs [26]. Manifestations of stigma driven by attitudes were observed frequently throughout the current study in the form of labeling, blame, scolding, and differential treatment.

##### Labeling

Labeling of AGYW seeking health services was commonly reported in both clinic and community settings. In the clinic, health workers reported observing fellow staff characterizing AGYW seeking SRH services as *‘a prostitute’, ‘sleeping around with older men’, ‘indulging’, ‘promiscuous’, or ‘mischievous.’* Health workers rarely described staff as saying these words to AGYW directly; rather, the labels were mostly described as either the health workers’ internal thoughts or as words expressed in conversations with colleagues.

Similar labels were reported as existing in the community. Health workers frequently described the community quickly assuming that AGYW who seek SRH services are *‘prostitutes’* or *‘promiscuous.*’ Although prostitution was by far the most commonly reported label, a few health workers discussed married women in the community also describing AGYW as a sexual threat who could be sleeping with their husbands or sons: *‘Of course … they’d even think these are the girls that are sleeping with our husbands.’*

##### Blame

These labels applied to AGYW were often accompanied by blame, which was reported in the clinic as comments or thoughts from staff evoking themes of intentionality and consequences of perceived misbehavior: *‘you’ve found the HIV that you were looking for.’* Some health workers mentioned the assumption that AGYW living with HIV intentionally infect others through sex; one health worker noted that some staff may treat AGYW differently based on this belief. Others stated that the amount of blame applied to AGYW living with HIV could depend on the assumed mode of HIV acquisition: one said that staff pity those who acquired HIV through vertical transmission but blame those who acquired HIV otherwise.

Health workers described blame in the community emerging in similarly accusatory language implying intentionality and fault, such as saying that AGYW who became pregnant or contracted HIV are *‘reaping what they are sowing.’* As observed in the clinic, AGYW living with HIV who were assumed to be sexually active faced heightened blame in the community as intentionally trying to spread the disease: *‘They are like Devils who want others to also fall sick because they are sick.’* In addition, one health worker discussed the variation in blame based on mode of HIV acquisition as also present in the community. In situations where AGYW acquired HIV vertically: *‘they will blame the parents, they will not blame the child.’* In situations where HIV acquisition was not vertical: *‘the blame is on herself.’*

##### Scolding

Health workers often reported observing scolding or chastising of AGYW who were perceived as sexually active, living with HIV, or seeking SRH services. In the clinic, this most commonly took the form of lecturing, shouting, or intrusive questioning with undertones of blame and judgment. When describing how staff view AGYW when they attend the clinic, health workers frequently mentioned that staff say that AGYW *‘do not listen’*, are *‘rushing’* into adulthood by *‘indulging’* in sex, and should be in school rather than the clinic. Some acknowledged that asking AGYW questions could stigmatize them even when it is well intentioned: *‘All those questions … may sound okay with you who is asking but the person that is getting those questions may feel bad.’*

The scolding words, phrases, and tone that health workers described in the clinic setting are reflective of what health workers described happening in the community towards AGYW. As in the clinic, community scolding includes condemning AGYW because they *‘do not listen’* or are *‘misbehaving’, ‘indulging’*, or *‘rushing.’*

##### Differential treatment

Health workers depicted differential treatment as a form of punishment in both the clinic and community for breaking social norms, although they described it as more frequent and explicit in the community. One health worker mentioned that when an AGYW comes into the clinic seeking SRH services, *‘some people will look at [it] as a very big issue like it’s a sin or it’s a bad thing to do, and overlook them and maybe maltreat them.’* Differential treatment in the clinic was also described as often based on assumed mode of HIV acquisition and marital status. As one health worker explained, those who acquired HIV through sexual transmission rather than birth are *‘treated badly automatically,’* while another reported that AGYW who come to the clinic without a husband will *‘be attended last.’*

In the community, health workers commonly reported that AGYW who became pregnant or were living with HIV faced isolation and rejection from their peer groups, relationships, and families. Some health workers described this isolation as imposed by the peers themselves, while others described the isolation as imposed by peers’ parents: *‘they will say she’ll teach her bad manners.’* This physical isolation from the community was sometimes reported as related to fear of contracting HIV. A few health workers reported hearing of partners leaving an AGYW after discovering she is living with HIV. A similar number of health workers described rejection from the AGYW’s own family, sometimes even through physical isolation within the home because the family fears contracting HIV.

#### Awareness as a driver

A second driver specified in the framework, awareness, acknowledges that individuals often do not consciously mean to stigmatize and are often unaware of the stigmatizing impact of their words or actions [26]. Manifestations of stigma driven by a lack of awareness of stigma emerged often throughout the interviews in the form of an awareness disconnect. Awareness disconnect appeared in both the clinic and community settings, specifically arising when respondents discussed the extent to which stigma exists within health workers themselves, as well as when discussing the extent to which stigma exists as a societal issue.

##### Existence of stigma within self

Health workers described general comfort with providing SRH care to AGYW, most often citing prevention of negative health outcomes and the sense of duty as a health worker as reasons for this comfort level: *‘My personal answer will always be, prevention is better than cure … it’s better we prevent them from undergoing those stress.’* Health workers seemed to express an overall intent to provide AGYW with quality care, free of stigma. Despite the presence of this intent, interviews indicated a remaining, though often unrecognized, presence of stigma within the health workers themselves.

Although infrequent, health workers sometimes expressed overtly stigmatizing thoughts about AGYW seeking SRH care. When asked if stigma was an issue for AGYW, one health worker answered: *‘It is an issue. Because in the first place they know that having sex before we get married is a crime in short … It is something wrong to do. So, for them to … access family planning, definitely it’s a clear picture to say, I am having sex. There’s stigma in that.’* This statement frames AGYW behavior, rather than the way AGYW are treated by the community and clinic, as the root issue of stigma.

More frequently, health workers’ statements revealed the presence of implicit stigma. Even after stating that AGYW should all be treated the same, health workers often distinguished levels of AGYW ‘guilt’ based on how they acquired their condition. This continuum of guilt was particularly common when discussing AGYW living with HIV. Health workers seemed to differentiate between those who acquired HIV at birth, those who acquired HIV after birth but are not perceived as to blame (those who were raped), and those who acquired HIV after birth and are perceived as to blame (those who were *‘indulging’* or *‘sleeping around’*): *‘Of course to those who [were] born with it sometimes some people they might think it is fine, let’s give this the priority even those who have been raped but those who acquired maybe sexually … the feeling might be different.’*

Implicit stigma also emerged in the health workers through generalized assumptions about AGYW motivations and circumstances. Some health workers described AGYW who attend the clinics as *‘dropouts’* who do not have a *‘zeal for school’* and find themselves *‘drinking beer’* or ‘*ever busy looking for money … [selling] their bodies for the exchange of money even without protection.’* In addition to money, health workers perceived the pressure to find a romantic partner as a primary motivator for sexual behavior. Health workers also made generalized assumptions about the AGYW’s family and upbringing, sometimes blaming AGYW behavior on the assumption that their parents are *‘drunkards’* or *‘prostituting.’*

Lastly, some health workers misunderstood the role of confidentiality breaches as a contributing factor to stigma. Through phrases such as *‘You know how us ladies are, we talk a lot,’* health workers implied that it was normal or to be expected for staff to talk about their patients. Other health workers seemed to separate confidentiality and public rebuke from the overall quality of the service:

‘Some they even talk like that but still even if they talk like that, they give the service.’

‘You shout [at] that person, but at the end they will just have to treat that person because it’s the service they were called to do.’

Despite frequent disconnect in the awareness of one’s own stigma, some health workers did acknowledge the influence of community norms, attitudes, and behaviors on their own levels of stigma. For example, some mentioned that regardless of their professional role as a health worker, they are humans who are members of a larger community: *‘You know at the end of the day the nurses, the health workers we are just human and with different attitudes, so it [can go] either way.’* Another shared, ‘*But of course even if they’re trained like that, the human nature in them will just comment ‘My daughter why?’’* Some clinical staff also stated explicitly that their parental caregiving roles influenced their views on providing care to AGYW: *‘We tend to, because maybe we have children … who have passed that age [or] are in school, so we want to compare our children to those ones that are coming.’*

##### Existence of stigma as a societal issue

Awareness disconnect was also reflected at the community level, as health workers sometimes demonstrated a contradictory lack of awareness when discussing the extent to which stigma was still a problem in society. Despite describing firsthand observation of stigma frequently in the community and to a lesser extent in the clinic, health workers often referenced the idea that stigma has decreased or does not exist anymore. Downplaying or denying stigma as an issue, with statements such as *‘Stigmatization now is not there in the community. I don’t feel it so much’* and *‘Lately because of the sensitisation that has been going around … I think everyone [has been] feeling free because we’ve been telling them to feel free,’* directly contradicts the detailed descriptions of existing stigma that health workers gave in other parts of the interview. This kind of awareness disconnect includes the idea that HIV stigma is less of an issue in recent times because it is easier to hide that one is living with HIV, which inherently implies that stigma still exists once HIV status is disclosed: *‘The good part is when you take your medication correctly … no one will label you [as] HIV positive.’*

Further, health workers often minimized the existence of societal stigma by referring to it as an issue primarily rooted in AGYW ‘self-stigma.’ This attitude portrays anticipated stigma as arising from AGYW themselves, rather than as a reflection of existing stigma from external sources. One health worker even implied that AGYW imagine the stigma they experience, in direct disconnect with health workers’ detailed descriptions of enacted stigma in both clinic and community settings: *‘You start imagining things that people are not even talking about you. That is why self-stigmatization [is] actually the worst. People haven’t even talked about what you are thinking you have already started thinking on their behalf.’*

#### Institutional environment as a driver

Although less prevalent than the other two drivers, institutional environment [26] also emerged as a driver, manifesting in the form of unwanted disclosure. Health workers recognized that AGYW face unwanted disclosure of their health conditions or reasons for visiting the clinic and described two institutional sources of unwanted disclosure: from staff who breach confidentiality and from the facility layout. Health workers gossip about their patients: when one health worker was asked about whether clinic staff discuss among themselves the AGYW who come in for family planning, they responded *‘… obviously (laughs).’* The clinic facility setup can also lead to breaches of confidentiality, particularly because shared waiting rooms lack privacy. Health workers frequently mentioned older married women asking the AGYW uncomfortable questions or gossiping to the community about an AGYW seen at the clinic: *‘Then the moment … the young girl leaves, they’d start talking to say, ‘Ah! I know that girl, she’s still in school, she’s very young but why is she getting family planning. Does the mother even know that this is what she does?’’.*

This unwanted disclosure of visits to the clinic reflects a general culture of gossip and lack of privacy in the community. Health workers reported general community gossip about a variety of AGYW clinic activities, including HIV treatment—*'whispering or gossiping among themselves ah you hear … even that one is taking yeah, so when we just hear that just know that meaning they mean ARVs’—*and family planning—*'the community will still talk, why having family planning at this age?’.*

### Locus of stigma

Although the interviews depicted common manifestations of AGYW stigma across both clinic and community levels, health workers often portrayed the locus of the stigma in different ways. Health workers often distinguished clinic stigma as dependent on the individual clinic staff member, while they tended to characterize community stigma as coming from the community as a whole. When asked how AGYW in the clinic are treated, health workers frequently responded that it depended on the person attending to them: *‘it depends on the nurse who is on duty, some nurse[s’] attitudes are not good, they [AGYW] are stigmatized.’* Sometimes health workers described differences in staff attitudes as based on AGYW age, marital status, or mode of HIV acquisition. Other health workers described these differences as stemming from variations in training or interest in adolescent-friendly care. Health workers seemed to perceive stigma in the clinics as individual based rather than systemic or institutional, which contrasted with the overarching nature of how they talked about stigma in the community.

### Impacts of stigma

Health workers clearly recognized the negative impacts of stigma for AGYW, both in the clinic and in the community. These negative impacts at several points across the care continuum—diagnosis, linkage to and retention in care, and treatment adherence—ultimately lead to negative impacts for AGYW health outcomes. Health workers acknowledged and frequently discussed anticipated stigma, reporting that AGYW are often afraid to access services because they anticipate negative attitudes from the adults in the clinic, including clinic staff and other patients: *‘They’re scared to meet with bigger people, those they know to say they’ll start blaming them, condemning them.’* Fear of being rebuked in long queues or public waiting rooms also often lead AGYW to wait to visit the clinic until their condition worsened: *‘[when] they come here they will probably be … in a bad state, they would have tried a lot of things at home but it will have failed.’* Particularly, health workers described the contribution of delayed antenatal care and unsafe abortions caused by stigma to maternal complications and mortality among AGYW.

Health workers also observed that many AGYW are hesitant to be truthful with their providers about their condition even when they have come to the clinic: *‘they don’t … directly say they have an STI. You know these guys are very (chuckles) … secretive. They’d come with a headache.’* Health workers further discussed the impact of stigma on retention in care, acknowledging that a negative first experience at the clinic can deter AGYW from returning for necessary follow-up appointments: ‘*If you treat an adolescent, the first treatment that you give will determine whether they’ll come back or not.’* Rather than going to the clinic, AGYW often prefer going to pharmacies, traditional healers, or friends for medical advice because these sources are perceived as more private or having less risk of stigma. Other health workers referenced the influence of anticipated stigma on ART adherence and resulting high viral loads: *‘We have seen a lot of children … they stop taking drugs, reason being that they don’t want their friend to know that they are taking drugs, so most of them when they come here … the viral load is very high.’*

## Discussion

To effectively address AGYW stigma in health facilities, it is necessary to understand stigma from the perspectives of both health workers and AGYW patients. This study illuminated health workers’ perceptions of the drivers, manifestations, and impacts of stigma against their AGYW patients, revealing that stigma observed in the clinic often mirrors stigma observed in the community. Although health workers seem to understand the negative impacts of stigma and often do not intend to stigmatize, they are still members of a larger community with deeply entrenched stigma, which can lead to unintended and unrecognized stigma in the clinic.

This study adds to the limited literature on health workers’ understanding of AGYW experiences and consequences of stigma across clinic and community settings [27]. It also supports existing literature on health workers’ own perceptions of AGYW care-seeking behavior, including the co-existence of cultural morality-based narratives surrounding premarital sex, which perpetuate health workers’ unfavorable attitudes and behavior towards AGYW seeking SRH services, and pragmatism related to adolescent health, which contributes to willingness to provide services [28–30]. The manifestations of AGYW stigma in the clinic described by the health workers in this study also echo the literature on the adolescent perspective, as adolescents have noted excessive questioning and scolding in health facilities as barriers to accessing care [10, 31]. Recent literature that explored youth-friendly clinic attributes of importance to Zambian AGYW also found that assurances of confidentiality and provider friendliness were key adolescent expectations when attending the clinic [32]. In addition, health workers’ comments about shared waiting rooms contributing to confidentiality concerns build on past acknowledgement of the role of the physical layout of the clinic facility as something that can contribute to or mitigate the experience of stigma [33].

Past literature has also quantitatively linked HIV-related stigma to negative impacts such as delayed care [11], decreased ART adherence [34], and lower retention in care [35], as recognized by the health workers in our study. AGYW hesitancy to disclose and concerns about privacy, identified by health workers as a stigma-related contributor to negative impacts in the current study, have been similarly discussed in previous research pointing to parental discouragement, anticipated stigma, and fear of rejection as barriers to disclosure [7, 36, 37].

This study offers insight that can be applied to increase the effectiveness of stigma-reduction programs in improving AGYW access to care. Amid greater focus on youth-friendly clinics in recent years, reviews of this literature have noted the universal importance of staff attitudes, including respect and friendliness, in encouraging young people to access health services [38, 39]. Despite the increasing amount of work in adolescent-friendly interventions [40], as well as health facility stigma-reduction interventions in general [14–19], there is limited work in understanding and developing adolescent-friendly interventions that incorporate facility-level stigma reduction. Recent literature discussing adolescent HIV stigma has noted the current lack of intervention literature in this area and called for additional focus on the drivers and impacts of health facility stigma when developing adolescent-friendly interventions [35, 41], a need also reflected by the continued existence of stigma as an often-named barrier to AGYW access to health services [22, 42, 43]. The remaining presence and impact of health workers’ negative behaviors and attitudes demonstrated in the current study emphasize the need for a specific focus on health facility stigma reduction as part of adolescent-friendly health services interventions.

The results presented in this study suggest that to be more effective, adolescent or youth-friendly interventions should add a specific stigma-reduction component that acknowledges and addresses the often unspoken disconnect arising between health workers’ clinic and community roles. The current study indicates that health workers are aware of stigma’s manifestations and negative impacts, recognize the importance of accessible SRH care, and, as a result, generally aim to avoid stigmatizing their patients. Yet at the same time, despite clearly describing the frequent manifestations and myriad harms of stigma, health workers’ statements often revealed a hesitance to acknowledge stigma as a societal issue or a lingering presence of stigma in the delivery of health services to AGYW. Although these contradictory statements are largely unintentional and unnoticed by health workers, they reflect the heavily intertwined nature of clinic-level and community-level stigma and continuing existence of implicit stigma in service delivery. The disconnect found in this study supports previous evidence that effective stigma-reduction interventions need to go beyond education [22]. Research has found that stand-alone stigma education programs can lead to stereotype suppression, rather than stereotype rejection, which in turn contributes to outward disapproval of stigma while failing to address the internal, deeply entrenched stigma that we all learn from society [44]. As seen in the interviews, health workers have learned what stigma looks like, understand that stigma in the clinic can lead to negative outcomes for AGYW, and largely intend to avoid stigmatizing AGYW. Although these findings suggest that health workers have learned to make an effort to suppress stigma, the disconnect that arose in their responses indicates that many may not have completely rejected stigma and that stigma may still influence care delivery. These results support further exploration of clinic-level stigma-reduction approaches that target awareness as a driver by moving beyond education to also include participatory, interactive opportunities that create a safe environment to self-reflect and discuss the roots of lingering implicit stigma [17].

Beyond the clinic level, these results echo previous calls for further research in multi-level interventions that address stigma across the socioecological model [22] and for research in how to integrate clinic- and community-based responses to adolescent HIV stigma [35]. In demonstrating that clinic-level stigma often reflects community-level stigma, this study indicates that clinic-level stigma does not exist in isolation. Rather, these interviews indicate that health workers’ stigma is rooted in societal stigma, suggesting that effective health facility stigma-reduction interventions need to incorporate the community level as well. This overlap in clinic- and community-level stigma indicates the need for continued focus on multi-level approaches to reduce HIV stigma against AGYW, building on current efforts such as the PrEPARE Pretoria Project [45] and the SHIELD intervention [23]. When designing stigma-reduction interventions for health workers, it is critical to explicitly recognize and address the influence of wider community stigma.

We acknowledge several limitations of note in this study. Social desirability bias, or the desire to overreport good things and underreport bad things in order to appear more favorable to others, could have influenced the health workers’ responses. Because health workers seemed to recognize the need to minimize stigma in clinics, their answers could have been biased by the desire to underplay the presence of stigma in themselves and their clinics. In addition, this study drew from a small sample of only six clinics, which may not be representative of a broader population of health workers. Our sample was also not large enough to identify whether there were differences in stigmatizing attitudes and awareness between clinical and non-clinical staff or between different lengths of employment in the field. Although the sample was small and purposively selected, saturation was achieved. Lastly, this study’s focus on the health worker perspective by nature lacks AGYW perspectives on the drivers, manifestations, and impacts of stigma in the clinic and community.

## Conclusions

Stigma is an oft-cited barrier to AGYW access to SRH services, including HIV prevention and treatment. As work continues to strengthen adolescent-friendly services in Zambia and beyond, adding or deepening a focus on addressing health facility stigma can improve health services delivery for AGYW. Addressing this stigma will require multi-level interventions that move beyond fostering knowledge about stigma and stated intentions to deliver stigma-free services to encouraging engaged critical thinking about internal beliefs and community influence and how these may manifest, often unconsciously, in service delivery to AGYW clients. These interventions should focus on closing the disconnect that emerged in the data through stigma awareness training to 1) build recognition of how AGYW stigma is manifesting in service delivery and its drivers, 2) deepen understanding of the myriad ways confidentiality can be breached in health facilities (e.g. gossip as a serious form of stigma, rather than behavior that is normal and expected), and 3) broaden understanding of the different sources and levels of stigma in the clinic as not just an issue of individual ‘bad actor’ health workers but as also emanating from deeply held beliefs rooted in the community. This study has implications for intervention adaptation, because it indicates that awareness-targeted and multi-level components should be incorporated into existing health stigma-reduction interventions [13, 14, 16–19, 46], and content specific to stigma reduction should be integrated into existing youth-friendly health services interventions [47–50].

## Supplementary Information

**Additional file 1.**

## Data Availability

Data and research findings generated from Eunice Kennedy Shriver National Institute of Child Health and Human Development–funded Prevention and Treatment through a Comprehensive Care Continuum for HIV-affected Adolescents in Resource Constrained Settings (PATC^3^H) studies will be made available in accordance with the National Institutes of Health Data Sharing Policy: https://grants.nih.gov/grants/policy/data_sharing/data_sharing_guidance.htm. The PATC^3^H program expects timely release and sharing of data generally coincident with publication of the study’s main findings no later than 1 year after acceptance of the PATC^3^H UH3 phase primary manuscript. The type of data made available must protect the rights and privacy of research participants. Data must be de-identified to the extent the sharing of data complies with Institutional Review Board guidelines along with local and/or United States law (whichever is more stringent) regarding the protection of patient privacy. Patient data that has been de-identified for sharing must not be able to be re-identified using publicly available data. Data for public use will be clean, de-identified study data submitted in DASH. The ‘public’ shall mean any requester of data outside of the PATC^3^H consortium. Data available to the public in the form of ‘public use’ data sets via DASH does not require a formal request to PATC^3^H for access, and hence, the PATC^3^H consortium is not responsible for the content of abstracts or manuscripts developed using these data. It is expected that publications and/or manuscripts that use PATC^3^H ‘public use’ data sets will acknowledge PATC^3^H. The interview guide is available as an additional file. The Dedoose codebook can also be made available to any requesters.

## Abbreviations

AGYW: Adolescent Girls and Young Women
ART: Antiretroviral Therapy
ARV: Antiretroviral
HIV: Human Immunodeficiency Virus
IDI: In-Depth Interview
SRH: Reproductive and Sexual Health
STI: Sexually Transmitted Infection
UNAIDS: The Joint United Nations Programme on HIV/AIDS
