# Supplementary material for: Health worker perceptions of stigma towards Zambian adolescent girls and young women: a qualitative study
Source: BMC Health Serv Res. 2022 Oct 17;22:1253. doi: 10.1186/s12913-022-08636-5 (PMC9575270; doi:10.1186/s12913-022-08636-5)
Supplement: Supplementary file 1 — Additional file 1. [file 12913_2022_8636_MOESM1_ESM.pdf]

Interviewer _______________Date: □□/□□□/□□ IDI number: □□□□□□□□□□□□□□

Understanding the role of stigma in restricting adolescent girls and unmarried young women's (AGYW) access to sexual and reproductive health services, as well as HIV testing and treatment

In-Depth Interview Guide: clinic staff

Copies of informed consent and confidentiality forms should be provided to each participant and read aloud for the benefit of those who cannot read. Participants should be provided an opportunity to ask any questions. Verbal agreement should be taped. The following is a guide. Try to ensure that all themes listed below are covered, while maintaining the flow of discussion. Suggested probes have been included under each topic area.

Note time interview starts.

Thank you for being willing to talk with me. Before we start, I would like to remind you that there are no right or wrong answers in this discussion. We are interested in knowing what you think, so please feel free to be frank and to share your point of view. It is very important that we hear your opinion. To begin with, I'd like to ask you a little bit about yourself.

Demographic info and work life of the respondent:

1. Please tell me about yourself and the work you do here at the clinic.
   a) Tell me a little bit about your position and what it involves on a daily basis.

2. *For non-clinical staff only:* In the course of your work, do you interact with adolescent girls (under age 18) and young women (ages 18-24) who come to the clinic? For example, by greeting them, directing them on where to go or registering them?
   a) Tell me a little bit about how you help adolescent girls and young women who come to this clinic.

3. *For clinical staff only:* In the course of your work, do you see/treat adolescent girls (under age 18) and unmarried young women (ages 18-24)? If so, what kind of services do you provide them with?
   a) *If they do not mention family planning or other reproductive health services:* What about family planning or other reproductive health services, e.g. STI screening or treatment? Do you ever provide these services to adolescent girls or unmarried young women?

4. *For clinical staff only:* Tell me a little bit about how family planning and other reproductive health services are organized in this clinic for adolescent girls (under 18 years) and unmarried young women (ages 18-24).
   a) Are there separate services (e.g. specific clinics or spaces/rooms where they are seen, or specific days/hours?) or are they integrated with other services?

- *If they do not mention them:* ask whether their facility has an adolescent corner.

5. *For clinical staff only:* What do you think about the way family planning and other reproductive health services for adolescent girls and unmarried young women are organized in this clinic?
   a) Would you change anything?
      - *If so*: What would you change? Why would you change it?

6. *For clinical staff only:* Have you received any training with regards to adolescent girls and young women's reproductive health?
   a) *If yes:* Please describe the training.
      - What did it cover?
      - How long was it?
      - What did you like about it?
      - What did you not like?
      - Were there any topics not included that you would like to learn about?
      - Who provided the training and how long was it?
      - Did the training include anything on stigma?
        - If yes, please describe the stigma piece of the training.

Theme 1: Pregnancy and STIs among adolescent girls and young women

Now I'd like to ask you about the situation of unmarried adolescent girls (under 18 years old) and unmarried young women (aged 18-24) in this community. From your own experience perhaps as a mother/father, auntie/uncle, or sister/brother, from what you have observed in the community, or what you have seen at health facilities, please…

1. Tell me a little bit about unmarried adolescent girls (girls under age 18) and unmarried young women (18-24) in this community. What are some of the challenges that they face?
   a) Is pregnancy an issue for them?
   b) What happens if an unmarried adolescent (under age 18) or unmarried young woman (ages 18-24) gets pregnant?
      - What do people say about her?
      - What do people say about her parents/family?
      - How is she treated in the community?
      - How about when she goes to a clinic for ANC? Is she treated (or received) any differently than adult married women? How? Why?

2. What about sexually transmitted infections? Are STIs an issue for unmarried adolescent girls (age under 18 years) and unmarried young women (ages 18-24) in this community?
   a) What would an adolescent girl or young woman do if she thinks she has an STI?
      - Who would she talk to?
      - Where would she go?
      - Would she go to a clinic?

- *If no:* why not?
- *If yes:* will she have any concerns about going to the clinic? Why?
    - How will she be received there by the staff? What will they say to her?
    - Will the staff have any concerns about offering her treatment?
    - Will they contact her parents before providing services?

3. What about HIV? Is HIV an issue for unmarried adolescent girls (age under 18 years) and young women (ages 18-24) in this community?
    a) What would an adolescent girl or young woman do if she thinks she has an HIV?
        - Who would she talk to?
        - Where would she go?
        - Would she go for an HIV test?
            - *If yes:* where would she go & why?
            - *If no:* why would she not go for a test?
                - Any other reasons?
    b) What are some of the challenges or barriers she would face in seeking HIV treatment in clinics in Lusaka? *(If they have already told you about challenges or barriers, modify to ask: "are there any other barriers or challenges that adolescent girls and young women face with respect seeking HIV treatment in clinics in Lusaka?")*

Theme 2: Provision of family planning/reproductive health services.

We have just discussed the general challenges adolescent girls and young women face in their communities, including around family planning and reproductive health services. So now I would like to ask you a bit about adolescent girls (under age 18 years) and unmarried young women's (age 18-24) utilization of family planning and other reproductive health services.

Depending on what they have told you already, adapt the phrasing of the above introduction to acknowledge what they have already shared. For example, could rephrase to: "You've just shared some of the issues adolescent girls and young women face in their communities around pregnancy and STIs. What are some of the challenges or barriers adolescent girls and young women face seeking services for these issues of family planning, STIs or other sexual and reproductive health services in clinics in Lusaka?"

1. Where do young girls in this community go to get family planning or other reproductive health services?
    a) Anywhere else?
    b) Do they come to this clinic?
    c) Do they prefer going one place more than another?
        - *If so:* Why?

2. How easy or hard is it for adolescent girls and unmarried young women to get family planning or other reproductive health services?
    a) Why?

   b) *Depending on answer & if not already answered:* What are some of the factors that help them get these services?
   c) *Depending on answer & if not already answered*: What are some of the factors that make it hard for them to access these services?

3. What do people in the community think or say about these girls, if they find out they are going for family planning or reproductive health services?

4. What do clinic staff think or say about these girls, when they find out they are at the clinic for family planning or reproductive health services?

5. In general, how do you think staff in this facility view these adolescent girls and unmarried young women?
   a) Are they comfortable providing them with family planning or other reproductive services?
      - Why or why not?
   b) If they are not currently providing family planning and reproductive health services to adolescent girls and unmarried young women as part of their daily work, how comfortable do you think your colleagues would be adding/integrating such services to their current work?
      - Why do you think this is?

6. What concerns do clinic staff have about providing family planning or reproductive health services to adolescent girls and unmarried young women?
   a) Any other concerns?
   b) *If no concerns raised:* Do clinic staff have any concerns about contraceptives and adolescent girls or young women's current or future health?
      - What are those concerns?
   c) Do clinic staff have any concerns about what the community will think or say if they find out that clinic staff have provided family planning and reproductive health services to adolescent girls?
      - What about to unmarried young women (aged 18-24?). Are the concerns different depending on the age of the unmarried girl?
        - Why?
   d) What about the families of the girls or young women? Do staff have any concerns about the parents or other family members finding out that they have provided these services to their adolescent daughters? Or their unmarried young women?

7. How often would you say adolescent girls or young women seek family planning or other reproductive health services in this facility?
   a) Tell me a little bit about these adolescent girls and young women.
      - What is their situation?
      - *If they say they don't come:* Why do you think they do not come here for services?

8. What about an adolescent girl or an unmarried young woman seeking family planning or reproductive health services here? How do you think she feels about coming here to seek these services?
    a) Why do you think she feels that way?
    b) What might be some of her hesitations, concerns, or fears?

9. Are adolescent girls and unmarried young women treated differently/received differently in any way than older women who are also seeking family planning or reproductive health services?
    a) *If yes*: in what way?

<u>Theme 3 (For clinical staff only): Facility Policy and Personal Provision of family planning/reproductive health services to young girls.</u>

Now I would like to ask you a bit about your own experience with providing adolescent girls and unmarried young women family planning and other reproductive health information and services.

1. Do you think current policies in your health facility are supportive of providing family planning/reproductive health information and services to adolescent girls and unmarried young women?
    a) Why or why not?

2. What about government policies and guidelines? What do they say?
    a) Do you know if they support adolescent girls and unmarried young women being given information on family planning?
        - *If yes:* Do the policies say anything about age at which family planning and other reproductive health services can be provided without parental or guardian consent?

3. What do you think about this? At what age to you think it should be allowed to provide family planning and reproductive health services to adolescent girls?

4. In your roles at this facility have you provided advice/information to adolescent girls and unmarried young women on family planning or other reproductive health services?
    a) *If yes:* What type of information did you provide?
    b) *If yes:* How did you feel about providing such information to adolescent girls under the age of 18 and unmarried young women (aged 18-24)?
        - How comfortable are providing family planning and other reproductive health services to adolescent girls under the age of 18?
            - What about unmarried young women aged 18-24?
        - Any concerns? Fears?
        - What would the community think about you doing this? What might they say about you?

- What about their parents? How would they react if they knew you had provided family planning or other reproductive health services to their daughters?

c) *If no:* What are the reasons you have never provided adolescent girls or young unmarried women with information on accessing family planning/reproductive health services?
    - Any other reasons? Concerns? Fears?
    - How comfortable would you be providing family planning and other reproductive health services to adolescent girls under the age of 18 in the future? What about unmarried young women aged 18-24?
        - *If comfortable*: why?
        - *If not comfortable*: what would prevent you from advising adolescent girls and unmarried young women to seek family planning and other reproductive health services?

5. How would you feel about having sexual and reproductive services for adolescent girls and young women integrated into the regular/routine services you provide, that is services that are not specifically targeted at adolescent girls and young women and are not reproductive health services?
    a) How well do you think integration would work?
    b) Would you have any concerns? What are they?
    c) Any suggestions of how best to integrate family planning and reproductive health services for adolescent girls and young women at this clinic?

<u>*Theme 4: Stigma*</u>

Now I'd like to ask you a bit about stigma towards unmarried adolescent girls (under age 18) and unmarried young women (ages 18-24).

Link back to the previous sections, where you asked about challenges for—if they mentioned stigma, you can reframe the introduction here and question 15 to "you mentioned earlier that stigma is a challenge for adolescent girls and young women. Now I'd like to ask you to tell me a bit more about that stigma."

1. Do you feel stigma is an issue for adolescent girls and unmarried young women <u>who are having sex?</u>
    a) Tell me a little more about the stigma. What does the stigma look like?
        - What do people say, what words do they use to describe these girls and young women?
        - How do they behave towards them?
        - Who is saying these things/stigmatizing them?

2. Do adolescent girls and unmarried young women seeking family planning/reproductive health services at this health facility or other health facilities experience stigma?
    a) How/what kinds of stigma? Please give me some specific examples.
        - Who is stigmatizing them?

        - Facility staff? Is it just the clinical staff or also the support staff like guards and receptionists? What about other, particularly adult, clients?
    - Do staff discuss or talk about adolescent girls and young women who come in for family planning or other reproductive health services amongst themselves?
        - *If yes:* What do they say to each other about these girls and young women?
        - *If yes:* What words do they use to describe these girls and young women?

3. Why do you think adolescent girls and unmarried young women who seek family planning/reproductive health information or services are stigmatized?

4. Is the stigma different for unmarried adolescent girls and unmarried young women depending on what service they are seeking? For example, would an adolescent girl or unmarried young women experience more or less stigma if they are pregnant and seeking ANC or during delivery versus seeking family planning or treatment for an STI?
    a) Why, or why not?

5. What about if an adolescent girl or unmarried young woman was having sex and using contraceptives to protect from an unwanted pregnancy or using condoms to protect herself from an STI?
    a) Would that change the stigma in any way?
        - Why, or why not?
        - In what way?

6. Do you feel stigma is an issue for girls who are living with HIV?
    a) Why or why not?
    b) *If yes:* what does stigma look like?
    c) What words are used to describe girls who are living with HIV?
    d) How do people behave towards girls living with HIV?
    e) Who is saying these things/stigmatizing them?

7. Is the stigma towards girls living with HIV who are having sex the same or different than the stigma towards girls who are having sex, but are not HIV positive?
    a) How?
    b) Why?

8. How about boys living with HIV? Do they also face stigma? Is the stigma the same or different as towards the girls?
    a) In what way?
    b) Why do you think it is different?

9. Some adolescent girls and unmarried young women acquired HIV perinatally (at birth), while others did not. As a result, there may be differences in the way we think about or treat an adolescent girl or unmarried young woman living with HIV, depending on how they acquired HIV. Have you ever observed a difference in the way adolescent girls and young women are viewed or received by health workers depending on how they are known or assumed to have acquired HIV?

a) In what way?
b) Why do you think that is the case?

Wrap-up

Before we end our conversation**…**

1. Is there anything else you'd like to share with me?
2. Are there any questions you would like to ask me?

Thank you very much for taking time to speak with me today and share your knowledge. We know you are very busy and are grateful for your cooperation.

Note time interview ends.
